# Interactome Analysis of Human Phospholipase D and Phosphatidic Acid-Associated Protein Network

**DOI:** 10.1016/j.mcpro.2022.100195

**Published:** 2022-01-08

**Authors:** Rebecca Elizabeth Kattan, Han Han, Gayoung Seo, Bing Yang, Yongqi Lin, Max Dotson, Stephanie Pham, Yahya Menely, Wenqi Wang

**Affiliations:** Department of Developmental and Cell Biology, University of California, Irvine, Irvine, California, USA

**Keywords:** PLD, PA, PJA2, SPHK1, S1P, CRAPome, Contaminant Repository for Affinity Purification, HCIP, high-confidence interacting protein, mTOR, mammalian target of rapamycin, PA, phosphatidic acid, PLD, phospholipase D, S1P, sphingosine-1-phosphatate, SAINT, Significance Analysis of INTeractome, SFB, S tag-Flag tag-SBP tag, SPHK1, sphingosine kinase 1, TAP-MS, tandem affinity purification coupled with mass spectrometry, TSC, total spectral count

## Abstract

Mammalian phospholipase D (PLD) enzyme family consists of six members. Among them, PLD1/2/6 catalyzes phosphatidic acid (PA) production, while PLD3/4/5 has no catalytic activities. Deregulation of the PLD-PA lipid signaling has been associated with various human diseases including cancer. However, a comprehensive analysis of the regulators and effectors for this crucial lipid metabolic pathway has not been fully achieved. Using a proteomic approach, we defined the protein interaction network for the human PLD family of enzymes and PA and revealed diverse cellular signaling events involving them. Through it, we identified PJA2 as a novel E3 ubiquitin ligase for PLD1 involved in control of the PLD1-mediated mammalian target of rapamycin signaling. Additionally, we showed that PA interacted with and positively regulated sphingosine kinase 1. Taken together, our study not only generates a rich interactome resource for further characterizing the human PLD-PA lipid signaling but also connects this important metabolic pathway with numerous biological processes.

## Highlights

• Defining the interactome of human phospholipase D enzymes and phosphatidic acid.
• PJA2 functions as an E3 ubiquitin ligase of phospholipase D1.
• Phosphatidic acid interacts with and positively regulates sphingosine kinase 1.

## Graphical Abstract

**Figure:**
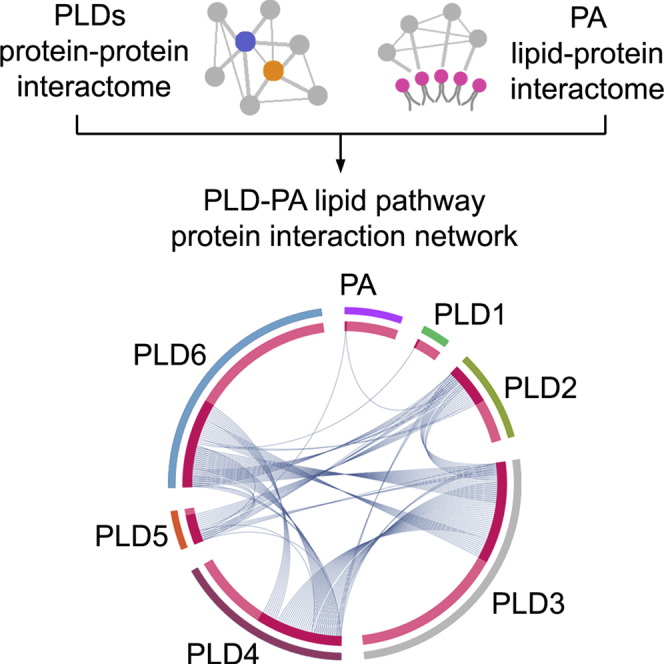

## In Brief

Using a proteomic approach, Kattan *et al.* defined the protein interaction network for the human phospholipase D family of enzymes and their lipid product phosphatidic acid and revealed diverse cellular signaling events involving this important lipid metabolic pathway.

The phospholipase D (PLD) enzyme family is known for its catalytic action to produce phosphatidic acid (PA) (1). Originally identified in *Salmonella typhimurium* (2), PLDs display conserved functions in numerous organisms (3). The mammalian PLD enzymes comprise six members (*i.e.*, PLD1–6), sharing the conserved HKD motif. The HKD motif contains the sequence HxKxxxxD (H: Histidine; K: lysine; D: Aspartic acid; x: any amino acid) and is essential for the catalytic activity of PLDs (4). Additional domains found in some PLD family members such as PH and PX domains are responsible for their membrane localization (5).

Among the human PLD enzymes, PLD1 and PLD2 are ubiquitously expressed in multiple tissues and play vital roles in various physiological processes, including receptor-mediated endocytosis, cell migration, and cytoskeletal organization (6, 7). Deregulation of these two PLD members has been associated with different human diseases (8, 9). PLD1 and PLD2 are 50% identical and share similar regulations and functions. For example, they both can be activated by ARF family GTPases (10, 11) and PI(4,5)P2 (12). PLD1 is localized in perinuclear structures such as Golgi, early endosomes, late endosomes, and lysosomes (6, 13, 14, 15), whereas PLD2 is found in the plasma membrane, exosomes, Golgi, and cytoplasm (16, 17). These findings suggest their roles in organelle-based cellular events. Additionally, PLD1/2 has been reported to regulate several growth-related signaling pathways through its lipid product PA such as the mammalian target of rapamycin (mTOR) (18) and the Hippo pathway (19), highlighting their crucial roles in growth control and cancer development. As for other PLD family members, PLD3 and PLD4 are endoplasmic reticulum (ER)–associated proteins with no classic catalytic activity (20, 21). Instead, PLD3 and PLD4 show 5′ exonuclease activity involved in inflammatory cytokine response (22). PLD5 is another PLD family member with no catalytic activity (23) and has been reported as an oncogene involved in prostate tumorigenesis (24). PLD6 is localized on mitochondria and regulates mitochondrial fusion (25). PA produced by PLD6 on the mitochondrial surface is converted to diacylglycerol to affect mitochondrial dynamics (26). PLD6 also plays a role in the Myc-mediated AMP-activated protein kinase activation, which in turn inhibits YAP/TAZ in the Hippo pathway (27).

As the major lipid product generated by PLD family enzymes, PA is found in cell plasma membrane and vesicles and has been extensively studied for its roles in vesicular trafficking, cytoskeletal dynamics, and endocytosis (28, 29). Interestingly, studies also revealed PA as a key signaling molecule involved in many signaling events *via* forming a complex with proteins. For example, PA directly binds and activates mTOR (30, 31). PA inhibits the Hippo pathway by interacting with its two components LATS and NF2 (19). MAPK cascade is regulated by PA through its physical interaction with Raf-1 (32). PA binds KIF5B to regulate membrane type-1 matrix metalloproteinase in cancer metastasis (33).

Pathologically, the PLD enzymes have been explored for their critical roles in numerous human diseases, including influenza (34), neurodegenerative disorders (35), autism (36), and fertility issues (26). In addition, enhanced PLD expression and activity are associated with many types of human cancer (37, 38, 39, 40), implicating their potential roles as biomarkers and targets for cancer treatment. Therefore, further characterizing the regulators and effectors for this key lipid enzyme family and its lipid product PA will not only provide novel insights into the PLD-PA lipid pathway in normal physiology but also reveal potential therapeutic targets for treating human diseases like cancer.

In this study, we conducted a large-scale proteomic study for the human PLD family enzymes and PA and defined the protein interaction network for this important lipid metabolic pathway. Through it, we connected the PLD enzymes and PA with various biological processes and discovered interacting proteins for PLDs and PA that may exert their cellular functions. Our functional studies further identified PJA2 as a novel E3 ubiquitin ligase for PLD1 and a negative regulator of mTOR signaling. Moreover, by characterizing the PA-sphingosine kinase 1 (SPHK1) lipid–protein complex, we established PA as a lipid regulator of SPHK1 to positively drive the SPHK1-dependent sphingosine-1-phosphate (S1P) production. Taken together, our interactome analysis of the PLDs and PA-associated protein interaction network provides a rich resource for further exploration of this key lipid metabolic pathway in various signaling events and biological processes.

## Experimental Procedures

### Cell Lines

HEK293T cells (a female cell line, ATCC: CRL-3216) were purchased from ATCC and kindly provided by Dr Junjie Chen (MD Anderson Cancer Center). HEK293A cells (a female cell line, Thermo Fisher Scientific: R70507) were purchased from Thermo Fisher Scientific and kindly provided by Dr Jae-Il Park (MD Anderson Cancer Center). HEK293A and HEK293T cells were maintained in Dulbecco’s modified essential medium supplemented with 10% bovine growth serum and 1% penicillin and streptomycin at 37 °C in 5% CO_2_ (v/v). Plasmid transfection was performed using a polyethylenimine reagent.

### Antibodies and Chemicals

For Western blotting, anti-Flag (M2) (F3165-5MG, 1:5000 dilution) and anti-α-tubulin (T6199–200UL, 1:5000 dilution) monoclonal antibodies were obtained from Sigma-Aldrich. An anti-Myc (sc-40, 1:500 dilution) monoclonal antibody was purchased from Santa Cruz Biotechnology. An anti-hemagglutinin (HA) monoclonal antibody (MMS-101P, 1:3000 dilution) was obtained from BioLegend. Anti-PJA2 (#40180, 1:1000 dilution), anti-MOB1 (# 3863S, 1:2000 dilution), and anti-phospho-p70 S6 kinase (Thr389) (#9234S,1:1000 dilution) polyclonal antibodies were obtained from Cell Signaling Technology. An anti-S1P (Z-P300; 1:500 dilution) monoclonal antibody was obtained from Echelon Biosciences.

For immunostaining, anti-HA (3724S, 1:3000 dilution) and anti-LAMP1 (9091S, 1:400 dilution) polyclonal antibodies were obtained from Cell Signaling Technology. An anti-Flag (F7425-.2MG, 1:5000 dilution) polyclonal antibody was purchased from Sigma-Aldrich. An anti-TOM20 (612278, 1:200 dilution) monoclonal antibody was purchased from BD Biosciences.

For chemicals, FIPI hydrochloride hydrate (#F5807) was obtained from Sigma-Aldrich. CAY10594 (#13207) was obtained from Cayman Chemical.

### Constructs and Viruses

Plasmids encoding the indicated genes were obtained from the Human ORFeome V5.1 library or purchased from DNASU Plasmid Repository. All constructs were generated *via* PCR and subcloned into a pDONOR201 vector using Gateway Technology (Thermo Fisher Scientific) as entry clones. For tandem affinity purification (TAP), all entry clones were subsequently recombined into a lentiviral gateway-compatible destination vector for the expression of *C*-terminal S tag-Flag tag-SBP tag (SFB)–tagged fusion proteins. Gateway-compatible destination vectors with the indicated SFB tag, HA tag, Myc tag, and mCherry tag were used to express various fusion proteins.

mCherry-Lysosomes-20 (plasmid #55073) and mCherry-ER-3 (plasmid #55041) were obtained from Addgene. PJA2 plasmid was kindly provided by Antonio Feliciello (University Federico II, Italy). RFP-PASS construct was kindly provided by Dr Guangwei Du (University of Texas Health Science Center at Houston).

PCR-mediated site-directed mutagenesis was used to generate amino acid mutations for PJA2 and SPHK1. PJA2 ring domain mutation was generated by mutating sites Cys634 and Cys671 to Ala (41). SPHK1 kinase dead (KD-5A) mutant was generated by mutating sites Ser165, Gly166, Asp167, Gly168, and Leu169 to Ala (42, 43, 44). The two SPHK1 hydrophobic patch mutants were generated by respectively mutating the site Leu280 to Ala and Phe283 to Ala/Leu284 to Gln (45).

All lentiviral supernatants were generated by transient transfection of HEK293T cells with the helper plasmids pSPAX2 and pMD2G (kindly provided by Dr Zhou Songyang, Baylor College of Medicine) and harvested 48 h later. Supernatants were passed through a 0.45-μm filter and used to infect cells with the addition of 8 μg/ml hexadimethrine bromide (Polybrene) (Sigma-Aldrich).

### Purification of PLDs and PA-Associated Protein Complexes

HEK293A cells stably expressing SFB-tagged PLD proteins were isolated by culturing in medium containing 2 μg/ml puromycin and validated by immunostaining and Western blotting as described (46). For tandem affinity purification, the HEK293A stable cells were lysed in 10 ml NETN buffer (100 mM NaCl; 20 mM Tris-HCl, pH 8.0; 0.5 mM EDTA; 0.5% Nonidet P-40) with protease and phosphatase inhibitors at 4 °C for 20 min. The crude lysates were centrifuged at 14,000 rpm at 4 °C for 15 min. The supernatants were incubated with 100 μl streptavidin-conjugated beads (GE Healthcare) at 4 °C for 6 h. The beads were then washed three times with 10 ml NETN buffer, and bound proteins were eluted with 1.5 ml NETN buffer containing 2 mg/ml biotin (Sigma-Aldrich) at 4 °C for 12 h. The elutes were incubated with 25 μl S protein beads (Novagen) at 4 °C for 4 h. The beads were then washed three times with 1 ml NETN buffer and subjected to sodium dodecyl sulfate–polyacrylamide gel electrophoresis. Each sample was run into the separation gel for a short distance, so that the whole bands could be excised as one sample for in-gel trypsin digestion and mass spectrometry analysis.

To isolate PA-associated protein complex, HEK293A cells were lysed in 10 ml NETN buffer with protease and phosphatase inhibitors at 4 °C for 20 min. The crude lysates were centrifuged at 14,000 rpm at 4 °C for 15 min. The supernatants were incubated with 30 μl control beads (#P-B000, Echelon Biosciences) or PA beads (#P-B0PA, Echelon Biosciences) at 4 °C for 12 h. The beads were then washed five times with 1 ml NETN buffer and subjected to sodium dodecyl sulfate–polyacrylamide gel electrophoresis. Each sample was run into the separation gel for a short distance, so that the whole bands could be excised as one sample for in-gel trypsin digestion and mass spectrometry analysis.

### Mass Spectrometry Analysis

The mass spectrometry was performed by the Taplin Mass Spectrometry Facility (Harvard Medical School) as described (46, 47). Briefly, the excised gel bands described above were cut into approximately 1-mm^3^ pieces. The gel pieces were then subjected to in-gel trypsin digestion and dried (48). Samples were reconstituted in 5 μl of HPLC solvent A (2.5% acetonitrile, 0.1% formic acid). The facility packed a nanoscale reverse-phase HPLC capillary column by packing 5-μm C18 spherical silica beads (Thermo Fisher Scientific) into a fused silica capillary (100 μm inner diameter × ∼20 cm length) with a flame-drawn tip. After the column was equilibrated, each sample was loaded onto the column *via* a Famos autosampler (LC Packings). A gradient was formed, and peptides were eluted with increasing concentrations of solvent B (97.5% acetonitrile, 0.1% formic acid).

As the peptides eluted, they were subjected to electrospray ionization and then entered into an LTQ Orbitrap Elite mass spectrometer (Thermo Fisher Scientific). The peptides were detected, isolated, and fragmented to produce a tandem mass spectrum of specific fragment ions for each peptide. Peptide sequences (and hence protein identity) were determined by matching protein databases with the fragmentation pattern acquired by the software program SEQUEST (ver. 28) (Thermo Fisher Scientific). Enzyme specificity was set to partially tryptic with two missed cleavages. Modifications included carboxyamidomethyl (cysteines, fixed) and oxidation (methionine, variable). Mass tolerance was set to 5 ppm for precursor ions and 0.5 Da for fragment ions. The mass spectrometry peak list was generated by ReADW.exe (version 4.3.1) using human as species. Peptide sequences were searched using Uniprot database (https://www.uniprot.org/proteomes/?query=taxonomy:9606) downloaded on June 20, 2017. The number of entries searched within this database are 160, 020 (half forward and half reverse and 168 contaminants added). Spectral matches were filtered to contain a false discovery rate of less than 1% at the peptide level using the target-decoy method (49), and the protein inference was considered following the general rules (50), with manual annotation based on experiences applied when necessary. This same principle was used for isoforms when they were present in the database. The longest isoform was reported as the match.

### Bioinformatic Analysis

As for the TAP coupled with mass spectrometry (TAP-MS) data analysis, a group of unrelated TAP-MS experiments (*i.e.*, 72 experiments using stably expressed SFB-tagged proteins as baits) were included as a control group. As for the PA beads-pulldown MS data, two control beads-pulldown MS data were included as a control group. Using the “sensitivity^1/2^-(1-specificity)^1/2^” measurement as determined by the Contaminant Repository for Affinity Purification (CRAPome) databases (51), we considered any interaction with a Significance Analysis of INTeractome (SAINT) score of at least 0.8 and raw spectra count of at least 2 to be a high-confidence interacting protein (HCIP) (46).

The Gene Ontology (GO) *p* values were estimated using metascape (www.metascape.org) (52), which contains findings and annotations from multiple sources including the GO database, KEGG pathway database, and Panther pathway database. Only statistically significant correlations (*p* < 0.05) are shown. The –log_10_ (*p* value) for each biological process with related HCIPs is listed.

### Experimental Design and Statistical Rationale

To apply SAINT analysis through CRAPome (https://reprint-apms.org/), we first gathered information about baits and preys including the spectra counts and assignment of control baits. We reorganized the data to the format compatible to the SAINT program and used two-pool analysis, which recognizes the control group as a separate pool. We did not remove outlier data points. The statistics used to assess accuracy and significance of measurements was referred to the SAINT algorithms, where SAINT score ≥0.80 was taken as the threshold required for the data quantification, as indicated by the SAINT method (53). In total, 6586 interactions were identified in 14 experiments, which includes biological replicates for each PLD enzyme and PA. There were a total of 74 unrelated negative control experiments that were used to carry out filtration *via* CRAPome. A total of 403 interactions passed a probability score of ≥0.80. To evaluate the specificity of our results, we overlapped our SAINT interactors among the six different isoforms/PA and considered the overlapped ones to be “false positives”, therefore designating 303 interactions as HCIPs. The PLDs and PA interactomes were generated using information from Ingenuity pathway software (Ingenuity Systems, www.ingenuity.com) for function/localization and Metascape (www.metascape.org) for GO. We used −log(*p* value) of individual functions to generate heatmap, where a *p* value <0.05 was considered statistically significant. The heatmap for the GO: biological processes and hierarchical clustering was generated by Rstudio.

### Immunofluorescent Staining

Immunofluorescent staining was performed as described (54). Briefly, cells cultured on coverslips were fixed with 4% paraformaldehyde for 10 min at room temperature and then extracted with 0.5% Triton X-100 solution for 5 min. After blocking with Tris-buffered saline with Tween 20 containing 1% bovine serum albumin, the cells were incubated with the indicated primary antibodies for 1 h at room temperature. After that, the cells were washed and incubated with fluorescein isothiocyanate, rhodamine, or Cy5-conjugated secondary antibodies for 1 h. Cells were counterstained with 100 ng/ml 4′,6-diamidino-2-phenylindole for 2 min to visualize nuclear DNA. The coverslips were mounted onto glass slides with an antifade solution and visualized under a Nikon Ti2-E inverted microscope.

### Gene Inactivation by CRISPR/Cas9 System

To generate the PJA2-knockout cells, five distinct single-guide RNAs were designed by CHOPCHOP website (https://chopchop.rc.fas.harvard.edu), cloned into lentiGuide-Puro vector (Addgene plasmid # 52963), and transfected into HEK293A cells with lentiCas9-Blast construct (Addgene plasmid # 52962). The next day, cells were selected with puromycin (2 μg/ml) for 2 days and subcloned to form single colonies. Knockout cell clones were screened by Western blotting to verify the loss of PJA2 expression. The oligo sequence information of single-guide RNAs used for knockout cell generation is listed in the supplemental Table S6.

### SPHK1 Kinase Assay

SFB-tagged SPHK1 was purified from serum-starved HEK293A cells using S protein beads; washed with NETN buffer, washing buffer (40 mM Hepes, 250 mM NaCl), and kinase assay buffer (30 mM Hepes, 50 mM potassium acetate, 5 mM MgCl_2_); and subjected to *in vitro* kinase assay in the presence of cold ATP (500 μM), sphingosine (1 mM), and PA (300 μM). The reaction mixture was incubated at 37 °C for 30 min and terminated with 1 M HCl. S1P was extracted with chloroform and dotted on nitrocellulose membrane. The S1P-dotted nitrocellulose membrane was blocked in 3% BSA at 4 °C overnight and followed by incubation with anti-S1P monoclonal antibody at 4 °C overnight.

To examine the roles of PLD inhibitors in regulating SPHK1 activity, SFB-SPHK1 was purified from HEK293A cells treated with dimethylsulfoxide, 30 μM FIPI, or 20 μM CAY10594 overnight using S protein beads, similarly washed as described above, and subjected to the kinase assay in the presence of cold ATP (500 μM) and sphingosine (1 mM). S1P dot blot assay was performed.

## Results

### Mapping the PLDs and PA-Associated Protein Network

**Fig. 1:**
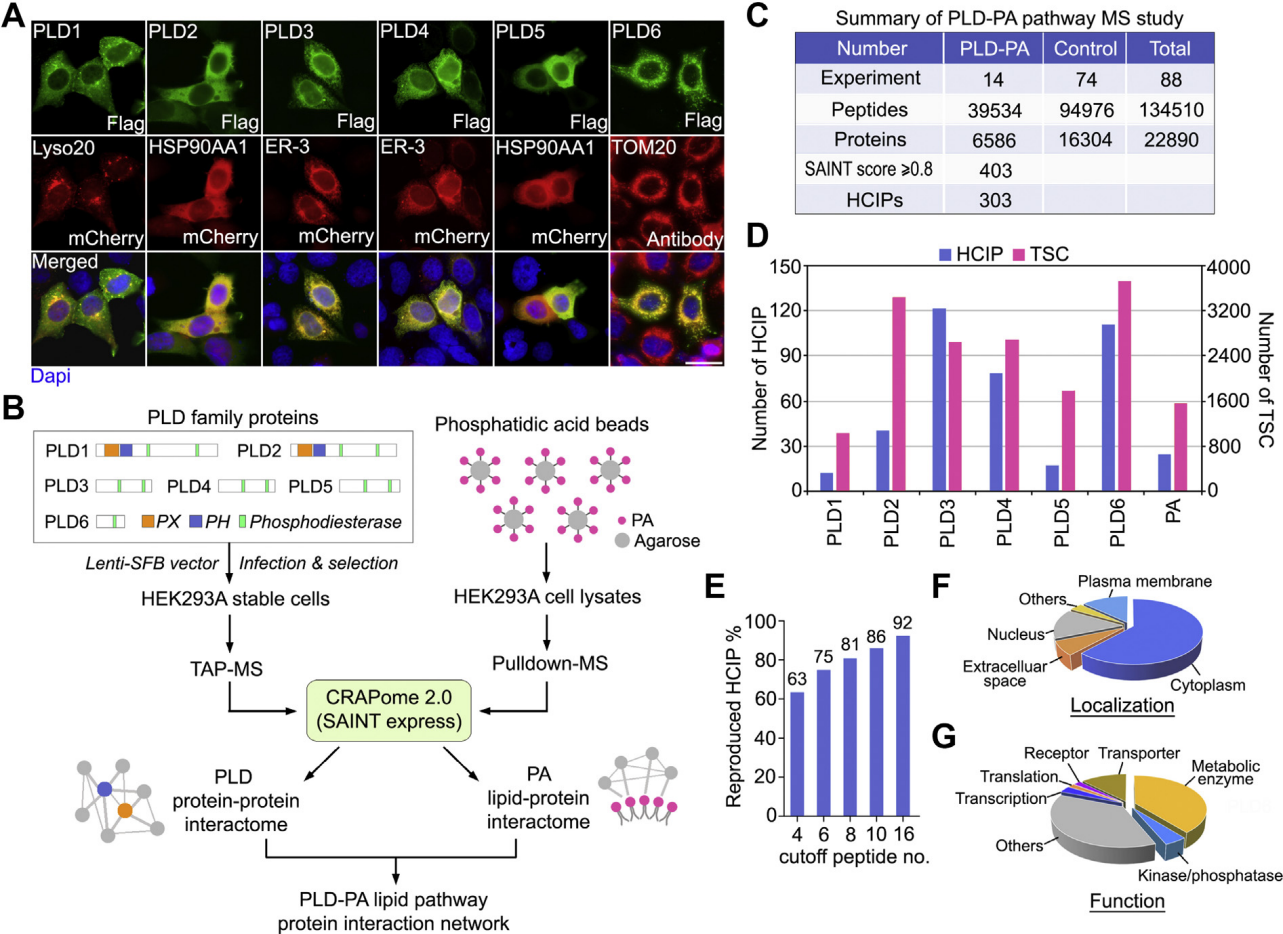
**Proteomic analysis of the phospholipase D (PLD) and phosphatidic acid (PA)-associated protein network.***A*, PLD family enzymes show different subcellular localizations. Immunofluorescent staining was performed using the indicated antibodies. Scale bar, 30 μm. *B*, schematic illustration of the major steps used in the proteomic study of human PLDs and PA-associated protein complexes. PLDs were constructed into a *C*-terminal SFB-tag fused lentiviral vector. HEK293A cells stably expressing each PLD bait protein were generated *via* lentiviral infection and puromycin selection and subjected to the TAP-MS analysis. For isolation of the PA-associated protein complex, HEK293A cells were subjected to pulldown assays using the PA-conjugated agarose beads. All the MS results were processed *via* the CRAPome 2.0 statistical model (*i.e.*, SAINT express). *C*, summary of the PLD-PA lipid pathway MS study. Experimental information and total numbers of peptides and proteins identified in the MS analysis were shown. A SAINT score ≥0.8 was used as the cutoff to identify HCIPs. *D*, the total spectral counts and corresponding numbers of HCIPs for PLDs and PA. *E*, Data reproducibility between two biological MS experiments for PLD members and PA are evaluated using the number of peptide spectrum matches. *F* and *G*, Gene Ontology annotations of the identified HCIPs of PLDs and PA. Cellular localization (*F*) and cellular functions (*G*) for the HCIPs of PLDs and PA were shown. HCIP, high-confidence interacting protein; SAINT, Significance Analysis of INTeractome; SFB, S tag-Flag tag-SBP tag; TAP-MS, tandem affinity purification coupled with mass spectrometry; TSC, total spectral count.

**Supplemental Figure S1:**
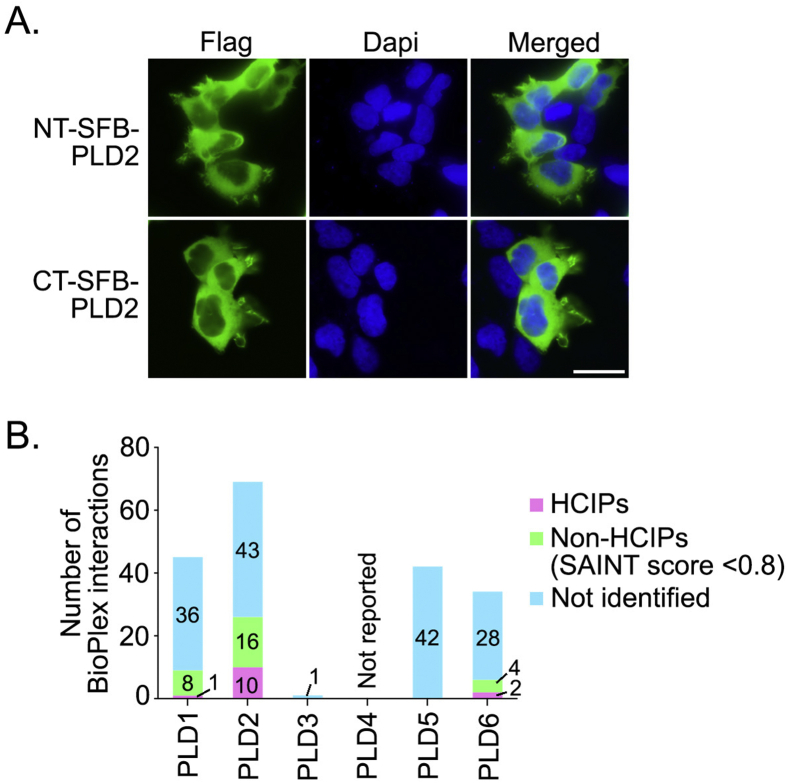
**Characterization of PLDs-based protein–protein interaction network.***A*, SFB-tag location does not affect PLD2 cellular localization. *N*-terminal SFB-tagged PLD2 and *C*-terminal SFB-tagged PLD2 constructs were expressed in HEK293A cells. Immunofluorescent staining was performed using Flag antibody. Scale bar, 30 μm. *B*, Comparison of the numbers of PLDs-interacting proteins identified in the BioPlex interactome database with the HCIPs of our dataset. HCIP, high-confidence interacting protein; PLD, phospholipase D; SFB, S tag-Flag tag-SBP tag.

To gain an overview of the human PLD family enzymes, we first examined their subcellular localization under normal culture condition. As shown in Figure 1*A*, a significant portion of PLD1 was localized on lysosomes, which is consistent with previous studies (13, 14, 15). PLD2 was majorly localized in the cytoplasm with partial membrane association (Fig. 1*A* and supplemental Fig. S1*A*). Similar to previous reports (20, 21), PLD3 and PLD4 were found as ER-localized proteins (Fig. 1*A*). In addition, previously uncharacterized PLD5 was found as a cytoplasmic protein, whereas PLD6 was mostly localized on mitochondria (Fig. 1*A*). These findings show distinct subcellular localizations for the human PLD enzymes.

To elucidate the intricate protein–protein interaction network for the PLD enzymes, we generated HEK293A cells stably expressing each of the six PLD members fused with a *C*-terminal SFB triple tags (*i.e.*, S tag-Flag tag-SBP tag) through lentiviral infection and puromycin selection (Fig. 1*B*). These PLD stable cells then underwent two independent TAP experiments after verification of bait protein expression and cellular localization. The associated proteins with each PLD enzyme were further examined using MS. In addition, the PA-bound proteins were isolated from HEK293A cell lysates using PA-conjugated agarose beads and analyzed by MS (Fig. 1*B*). A complete list of the peptides and proteins identified in this study can be found in supplemental Tables S1 and S2, respectively.

To refine the identified PLDs-interacting proteins, a group of 72 unrelated TAP-MS experiments performed under similar purification conditions were included as controls. Moreover, two independent pulldown experiments using agarose beads only were performed as controls for the identified PA-interacting proteins. We assigned a SAINT score to each identified hit and considered any interaction with a SAINT score at least 0.80 and raw spectra counts at least 2 as a HCIP (Fig. 1*B*). Through this filtration, a total of 303 HCIPs among the 6586 preys were identified for the PLD-PA lipid pathway (Fig. 1*C* and supplemental Table S3). The HCIP number and total spectral count number for each PLD member and PA were summarized in Figure 1*D*. Data reproducibility by comparing the two biological repeats for the PLDs and PA proteomic studies suggested the reliability of the dataset (Fig. 1*E*). GO analysis indicated that the identified HCIPs of PLDs and PA are widely distributed with different subcellular localizations and are involved in numerous cellular functions (Fig. 1, *F* and *G* and supplemental Table S3). Because the cells used for our proteomic study were grown under normal culture condition, the identified HCIPs should be considered as the basal-state interactome for the human PLD-PA lipid pathway. In addition, our proteomic study was performed in HEK293A cells, thus it may not recapitulate specific interacting proteins and unique cellular functions for some PLD proteins in specialized cells.

We next compared our PLDs HCIP list with the data reported in the BioPlex Interactome database (https://bioplex.hms.harvard.edu). Of 45 protein interactions identified for PLD1 in BioPlex, only one was identified as HCIP in our PLD1 interactome study (supplemental Fig. S1*B* and supplemental Table S4). Of 69 interactions identified for PLD2 in BioPlex, ten were identified as HCIPs in our PLD2 interactome study (supplemental Fig. S1*B* and supplemental Table S4). Of 34 interactions identified for PLD6 in BioPlex, two were identified as HCIPs in our PLD6 interactome study (supplemental Fig. S1*B* and supplemental Table S4). Actually, several of the interactions reported in BioPlex were also identified in our dataset but did not pass the stringent filtering criteria used in our study (eight interactions for PLD1, 16 interactions for PLD2, and four interactions for PLD6). In addition, the PLD3 interactome study in BioPlex only reported one interaction that was not identified in our dataset (supplemental Fig. S1*B* and supplemental Table S4). The interactome analyses of PLD4 and PLD5 were not included in BioPlex, although PLD5 was revealed as a prey protein in some interactome studies there (supplemental Fig. S1*B* and supplemental Table S4). Moreover, we identified additional high-confidence interactions with PLD proteins that were not reported in BioPlex, further extending our knowledge of the PLDs-associated protein network. Taken together, these results suggest that our PLDs proteomic dataset not only reproduced previous findings but also provided an additional collection of candidate HCIPs for further validation.

### Overview of the Protein Interaction Landscape for the PLD-PA Lipid Pathway

**Fig. 2:**
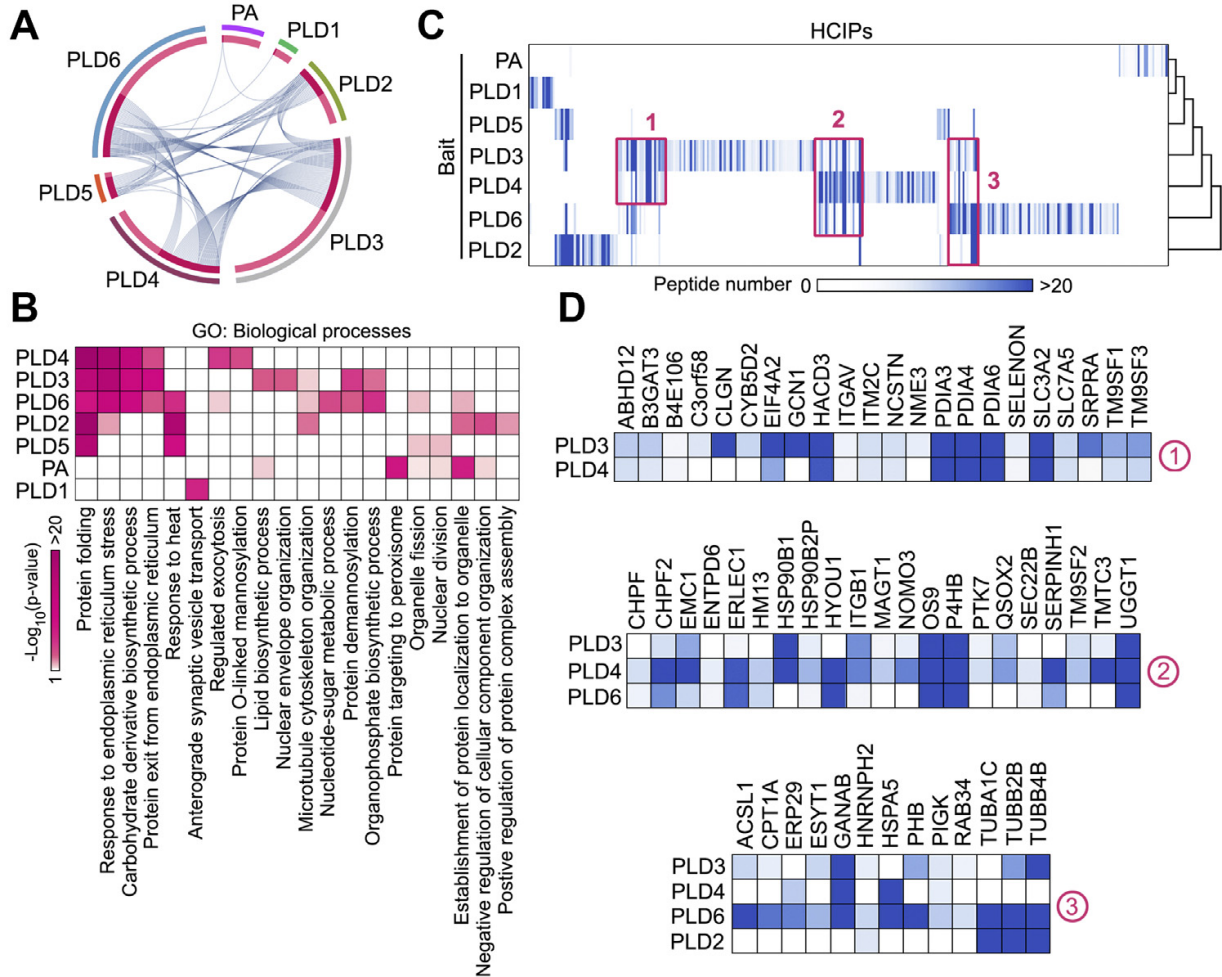
**Hierarchical clustering analysis of the HCIPs generated for the PLD-PA lipid pathway.***A*, the HCIPs shared between PLDs and PA were analyzed by www.metascape.org. *B*, the HCIPs of PLDs and PA were subjected to GO analysis. Through it, the biological processes of the PLDs/PA–HCIPs were shown as a heatmap. *C* and *D*, a heatmap was generated from hierarchical clustering of 303 HCIPs for PLDs and PA. Three prominent HCIP clusters were manually selected (*C*) and enlarged below (*D*). The color of *squares* in the heatmap indicates the identified HCIP peptide number for each bait. HCIP, high-confidence interacting protein; PA, phosphatidic acid; PLD, phospholipase D.

Interestingly, metascape analysis revealed that many HCIPs were shared among PLD family enzymes like PLD3, PLD4, and PLD6, while this was not the case for PLD1 or PA (Fig. 2*A*). We also connected the HCIPs of PLDs and PA with various biological processes *via* GO analysis. As shown in Figure 2*B* and supplemental Table S5, although the HCIPs of PLDs and PA were functionally categorized into multiple biological processes, they were highly enriched in protein modification, metabolism, and organelle-based cellular functions. As for each PLD member, the HCIPs of PLD3 and PLD4 were majorly involved in protein folding and ER-associated functions (Fig. 2*B*), which was consistent with the ER localization of these two PLD members (Fig. 1*A*). PLD3 also played roles in lipid biosynthesis and nuclear envelop organization, while PLD4 specifically regulated exocytosis (Fig. 2*B*), suggesting their unique functions through their binding proteins. Although localized on mitochondria (Fig. 1*A*), PLD6 could play a role in ER-related cellular events, because its HCIPs shared some biological processes with those of PLD3/4 (Fig. 2*B*). In addition, this GO analysis suggested roles of PLD1 and PA in synaptic vesicle transport and vesicle protein recruitment, respectively (Fig. 2*B*).

Based on these findings, we further examined the PLDs and PA-associated protein interaction network through a prey-oriented hierarchical clustering analysis. As shown in Figure 2*C*, some common subsets were revealed between different PLD family members. For example, PLD3 and PLD4 interacted with a wide range of ER proteins such protein disulfide-isomerase family members PDIA3/4/6 (cluster 1) (Fig. 2*D*). Additionally, PLD3, PLD4, and PLD6 shared HCIPs involved in ER protein folding, including OS9, P4HB, CHPF2, HYOU1, UGGT1, GANAB (cluster 2 and cluster 3) (Fig. 2*D*). These findings suggested that PLD3, PLD4, and PLD6 may play roles in ER-associated protein quality control and stress response. Interestingly, both PLD3 and PLD4 interacted with a group of lysosome-associated transmembrane proteins TM9SF1-3 (cluster 1 and cluster 2) (Fig. 2*D*), suggesting potential roles of PLD3 and PLD4 in mediating the crosstalk between ER and lysosomes. Moreover, PLD2 and PLD6 were strongly associated with tubulin subunits (*i.e.*, TUBA1C, TUBB2B, and TUBB4B) (cluster 3) (Fig. 2*D*), indicating that their functions may involve the microtubule-based events. Again, we hardly identified significant HCIPs clusters of PLD1 or PA with other PLD members (Fig. 2*C*), consistently showing relatively unique binding proteins for PLD1 and PA.

**Fig. 3:**
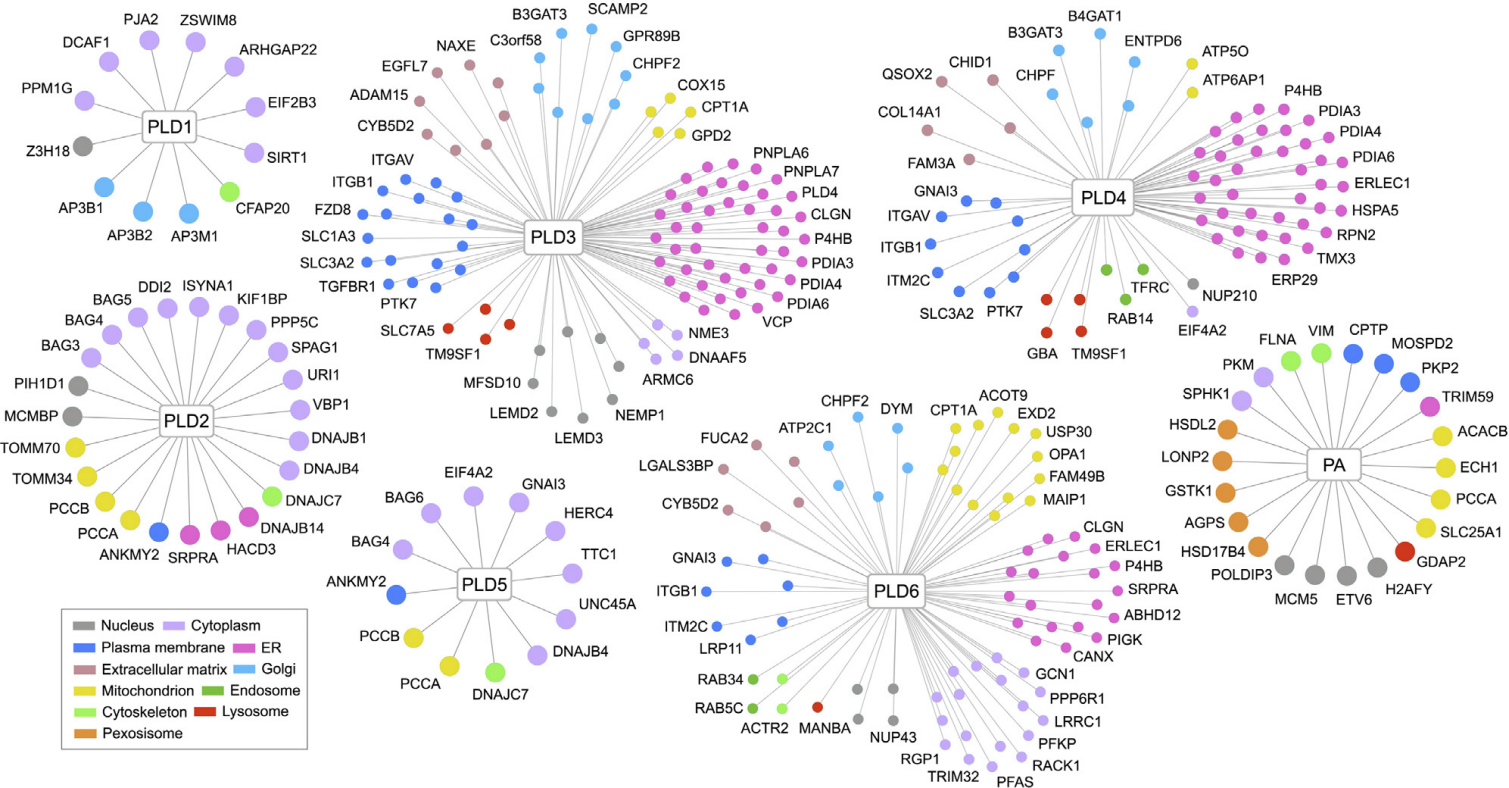
**Interaction maps of the human PLDs and PA-centered protein interaction network.** As for each PLD family member and PA, HCIPs are grouped based on their cellular localization according to the GO analysis and literature search and visualized in different colors. HCIP, high-confidence interacting protein; PA, phosphatidic acid; PLD, phospholipase D.

**Supplemental Figure S2:**
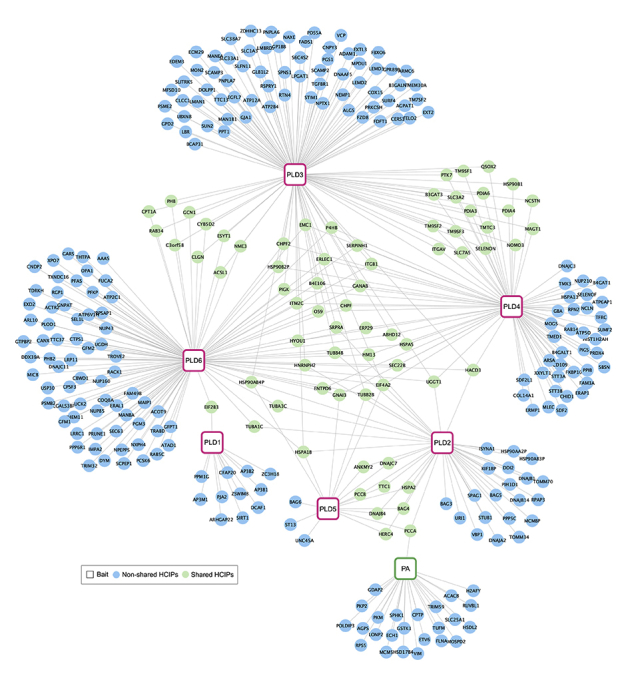
**Interactome map of the PLDs and PA-associated proteins.** The cytoscape-generated merged interaction network for six PLD proteins and their lipid product PA showed a group of shared HCIPs (*green*) between PLD proteins and PA. Unique HCIPs for each PLD protein and PA are labeled in *blue*. HCIP, high-confidence interacting protein; PA, phosphatidic acid; PLD, phospholipase D.

Next, we organized the PLDs and PA-associated protein network individually and functionally by clustering the HCIPs based on their cellular localization (Fig. 3). This comparative interaction network organization highlighted the different groups of HCIPs for each PLD member and PA and implicated their roles in the related biological processes. We also compared the HCIPs for individual PLD enzyme and PA and revealed a group of HCIPs as shared binding proteins among PLDs and PA (supplemental Fig. S2), which is consistent with the prey-oriented hierarchical clustering analysis (Fig. 2*C*). Taken together, these results show that PLD enzymes and PA share common and unique binding proteins, implicating their both redundant and specific functions within various cellular events.

### Validation of the PLD3/4/6-Associated Protein Interaction Network

**Fig. 4:**
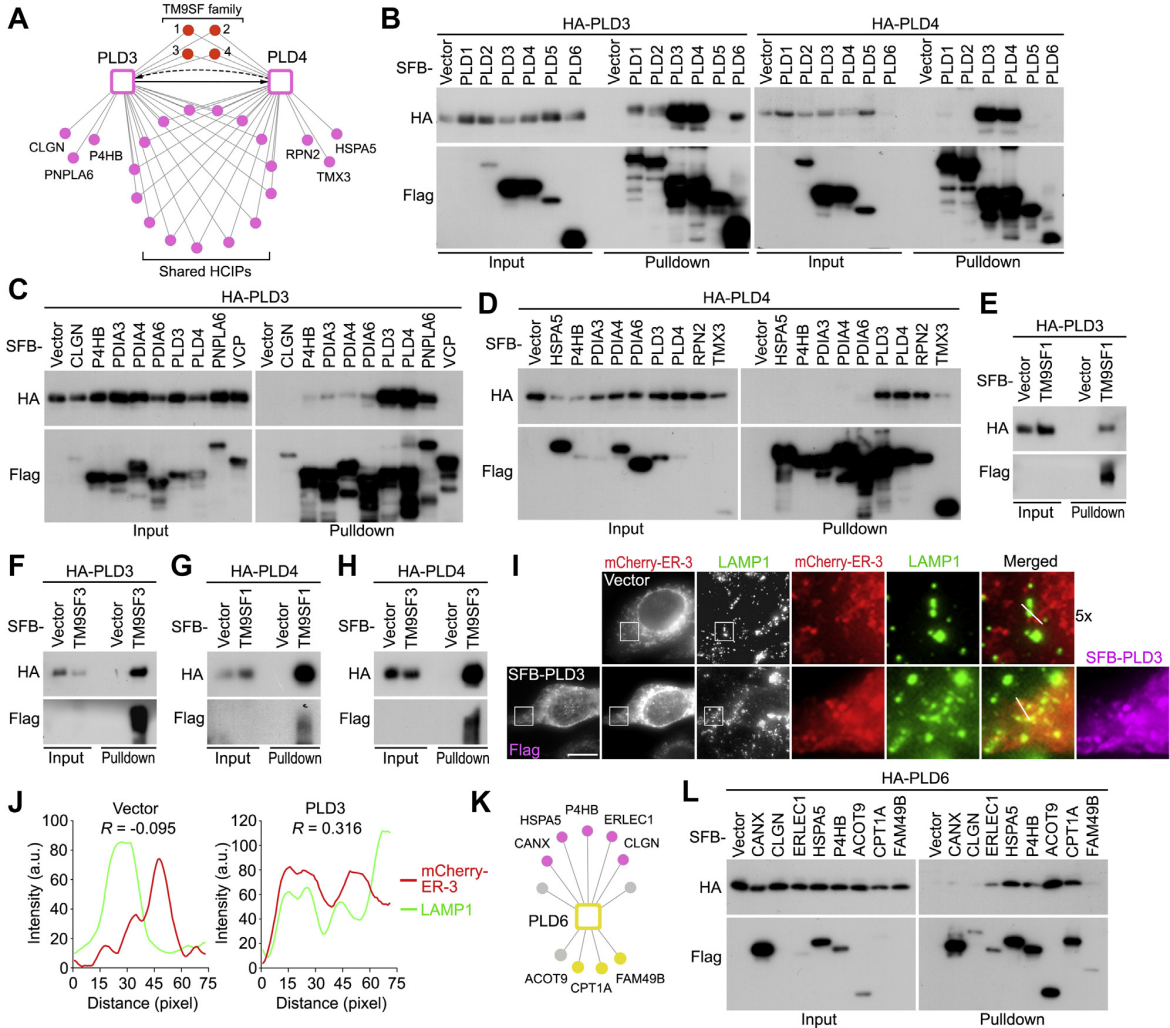
**Validation of the PLD3/PLD4/PLD6-associated protein network.***A*, a summary of the protein–protein interaction network with the selected HCIPs of PLD3 and PLD4. *B*, validation of the interaction between PLD3 and PLD4 in the PLD family. HEK293T cells were transfected with the constructs encoding the indicated proteins and subjected to pulldown assay using S protein beads. *C*, validation of the interaction between PLD3 and its selected HCIPs. HEK293T cells were transfected with the constructs encoding the indicated proteins and subjected to pulldown assay using S protein beads. *D*, validation of the interaction between PLD4 and its selected HCIPs. HEK293T cells were transfected with the constructs encoding the indicated proteins and subjected to pulldown assay using S protein beads. *E*–*H*, PLD3/4 interact with TM9SF1/3. HEK293T cells were transfected with the constructs encoding SFB-tagged TM9SF1 or TM9SF3 and HA-tagged PLD3 (*E* and *F*) or PLD4 (*G* and *H*) and subjected to pulldown assay using S protein beads. *I* and *J*, PLD3 drives ER-lysosome contact formation. Immunofluorescent staining was performed using the indicated antibodies (*I*). The indicated area were five times enlarged (5×). Scale bar, 15 μm. The fluorescent intensity correlation (R) between ER-3 (*red*) and LAMP1 (*green*) were analyzed (*J*) along the lines as indicated in (*I*). *K*, a summary of the protein–protein interaction network for the selected HCIPs of PLD6. *L*, validation of the interaction between PLD6 and its selected HCIPs. HEK293T cells were transfected with the constructs encoding the indicated proteins and subjected to pulldown assay using S protein beads. ER, endoplasmic reticulum; HCIP, high-confidence interacting protein; PLD, phospholipase D; SFB, S tag-Flag tag-SBP tag.

By examining the PLD3- and PLD4-associated proteins (Fig. 3 and supplemental Table S3), we found that they reciprocally identified each other (Fig. 4*A*). Indeed, pulldown experiments showed that PLD3 and PLD4 not only strongly interacted with each other but also bound their own, among the six PLD members (Fig. 4*B*). In addition to some shared HCIPs, PLD3 and PLD4 also specifically interacted with some ER proteins (Fig. 4*A*). Among them, the PLD3-PNPLA6 (Fig. 4*C*) and PLD4-RPN2/TMX3 (Fig. 4*D*) complexes were validated through pulldown assays.

Notably, several lysosome-associated TM9SF family proteins (*i.e.*, TM9SF1–4) were identified as shared binding partners for PLD3 and PLD4 (Fig. 4*A*). Among them, the associations of TM9SF1 and TM9SF3 with PLD3 (Fig. 4, *E* and *F*) and PLD4 (Fig. 4, *G* and *H*) were experimentally confirmed. Because TM9SFs and PLD3/4 are transmembrane proteins localized on lysosomes and ER, respectively, their interaction could play a role in ER-lysosomes contact formation. Interestingly, overexpression of PLD3 but not vector control significantly promoted the co-localization between ER and lysosomes as visualized by mCherry-ER-3 and LAMP1, respectively (Fig. 4, *I* and *J*). These data implicate a potential role of the PLD3/4–TM9SFs complex in mediating the contact formation between ER and lysosomes.

Unexpectedly, although PLD6 is majorly localized on mitochondria (Fig. 1*A*), our proteomic study revealed multiple ER proteins as its binding proteins (Fig. 3). Indeed, among the tested HCIPs (Fig. 4*K*), the association of PLD6 with ER proteins ERLEC1, HSPA5, and P4HB was confirmed through pulldown assays (Fig. 4*L*), which was comparable to that between PLD6 and two mitochondrial proteins ACOT9 and CPT1A (Fig. 4*L*). Thus, these data indicate a potential role of PLD6 in ER-related functions, which deserves further investigation.

### Characterization of the PLD1/2/5-Based Protein Interaction Network Uncovers PJA2 as an E3 Ubiquitin Ligase for PLD1

**Fig. 5:**
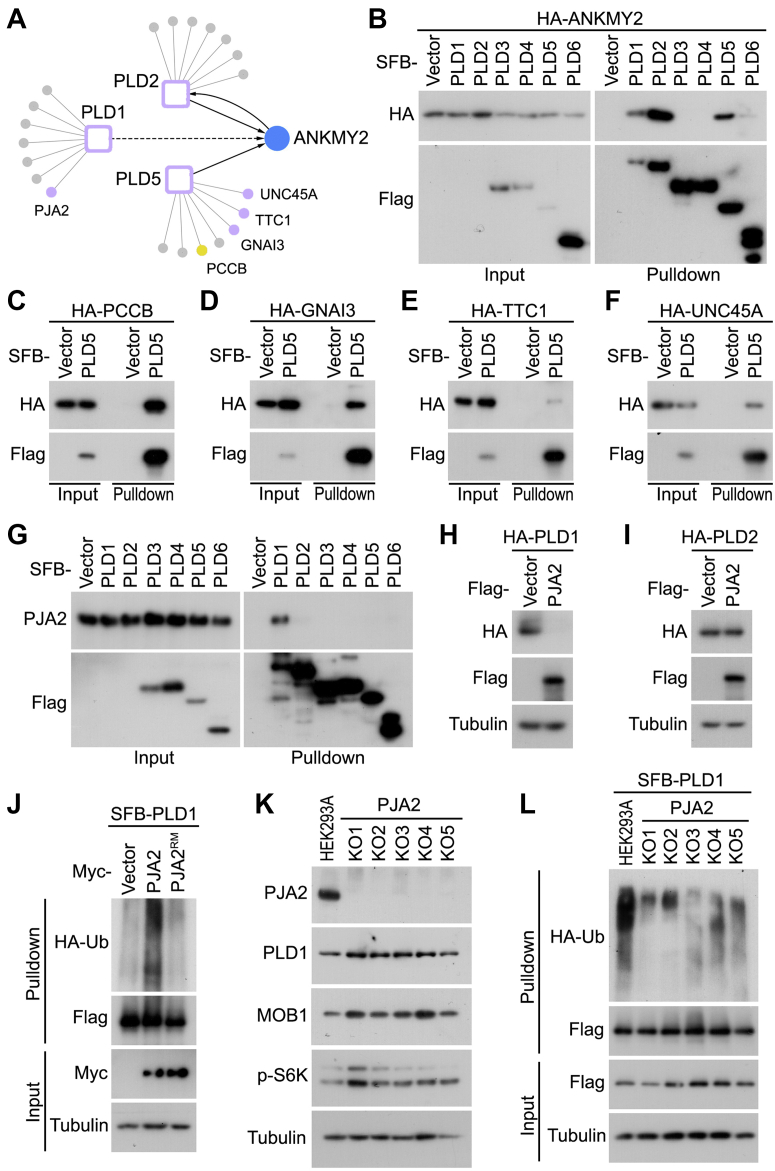
**Characterization of PLD1/PLD2/PLD5-associated protein network reveals PJA2 as an E3 ubiquitin ligase for PLD1.***A*, a summary of the protein–protein interaction network with the selected HCIPs of PLD1/2/5. ANKMY2 was indicated as the shared HCIP for these three PLD proteins. *B*, ANKMY2 interacts with PLD1/2/5. HEK293T cells were transfected with the constructs encoding the indicated proteins and subjected to pulldown assay using S protein beads. *C*–*F*, validation of the interaction between PLD5 and its selected HCIPs. Constructs encoding SFB-tagged PLD5 and HA-tagged PCCB (*C*), GNAI3 (*D*), TTC1 (*E*), and UNC45A (*F*) were transfected into HEK293T cells. Pulldown experiments were performed using S protein beads. *G*, PJA2 specifically interacts with PLD1 among the PLD members. HEK293T cells were transfected with the constructs encoding the indicated proteins and subjected to pulldown assay using S protein beads. *H* and *I*, PJA2 targets PLD1 protein expression but does not affect that of PLD2. Constructs encoding Flag-tagged PJA2 and HA-tagged PLD1 (*H*) or PLD2 (*I*) were co-transfected into HEK293T cells. Western blots were performed using the indicated antibodies. *J*, overexpression of PJA2 but not its ring domain mutant (RM) induces the ubiquitination of PLD1. HEK293T cells were transfected with the constructs encoding the indicated proteins, treated with MG132 (20 μM) for 8 h, and subjected to pulldown assay using S protein beads. Western blots were performed using the indicated antibodies. *K*, PLD1 protein expression is increased in the PJA2 knockout (KO) HEK293A cells. Western blots were performed using the indicated antibodies. *L*, the ubiquitination of PLD1 is decreased in the PJA2 KO HEK293A cells. The indicated HEK293A cells were transfected with the constructs encoding the indicated proteins, treated with MG132 (20 μM) for 8 h, and subjected to pulldown assay using S protein beads. Western blots were performed using the indicated antibodies. HCIP, high-confidence interacting protein; PLD, phospholipase D; SFB, S tag-Flag tag-SBP tag.

Upon analyzing the HCIPs of PLD5, we identified a Hedgehog pathway regulator ANKMY2 (55) as a shared interacting protein with PLD1 and PLD2 (Fig. 5*A*). Although pulldown experiments confirmed the interaction between PLD1/2/5 and ANKMY2 (Fig. 5*B*), PLD2 strongly interacted with ANKMY2 as compared to PLD1 and PLD5 (Fig. 5*B*). This result was consistent with the observation that PLD2 was the only PLD member identified in the ANKMY2 reverse TAP-MS study (Fig. 5*A*). In addition to ANKMY2, several PLD5 HCIPs, including PCCB (Fig. 5*C*), GNAI3 (Fig. 5*D*), TTC1 (Fig. 5*E*), and UNC45A (Fig. 5*F*), were further validated for their association with PLD5 through pulldown assays. These results suggest a variety of cellular events that may involve PLD5, providing functional insights into this previously uncharacterized PLD member.

PLD1 plays a key role in PA production, and dysregulated PLD1 expression has been associated with many oncogenic signaling events and observed in various human cancers (9, 37, 39). Interestingly, an E3 ubiquitin ligase PJA2 was identified as a binding partner for PLD1 (Figs. 3 and 5*A*). Therefore, we further examined whether PJA2 could target PLD1 protein stability and modulate its associated signaling events. Indeed, PJA2 specifically interacted with PLD1 among the six PLD members (Fig. 5*G*). Overexpression of PJA2 reduced PLD1 protein expression (Fig. 5*H*) but did not affect that of PLD2 (Fig. 5*I*). Moreover, expression of PJA2, but not its ring domain mutant PJA2^RM^, dramatically induced PLD1 ubiquitination (Fig. 5*J*). On the other hand, depletion of PJA2 stabilized PLD1 and another known PJA2 substrate MOB1 (41) (Fig. 5*K*) and also largely reduced the ubiquitination of PLD1 (Fig. 5*L*). Given the critical role of PLD1 in activating mTOR signaling (18), we examined whether PJA2 could modulate mTOR activity. Indeed, as shown in Figure 5*K*, loss of PJA2 promoted the mTOR-dependent S6 kinase phosphorylation. Taken together, these data demonstrate that PJA2 serves as an E3 ubiquitin ligase for PLD1 and controls PLD1 downstream signaling events.

### Analysis of the PA Interactome Reveals PA as a Positive Regulator of SPHK1

**Fig. 6:**
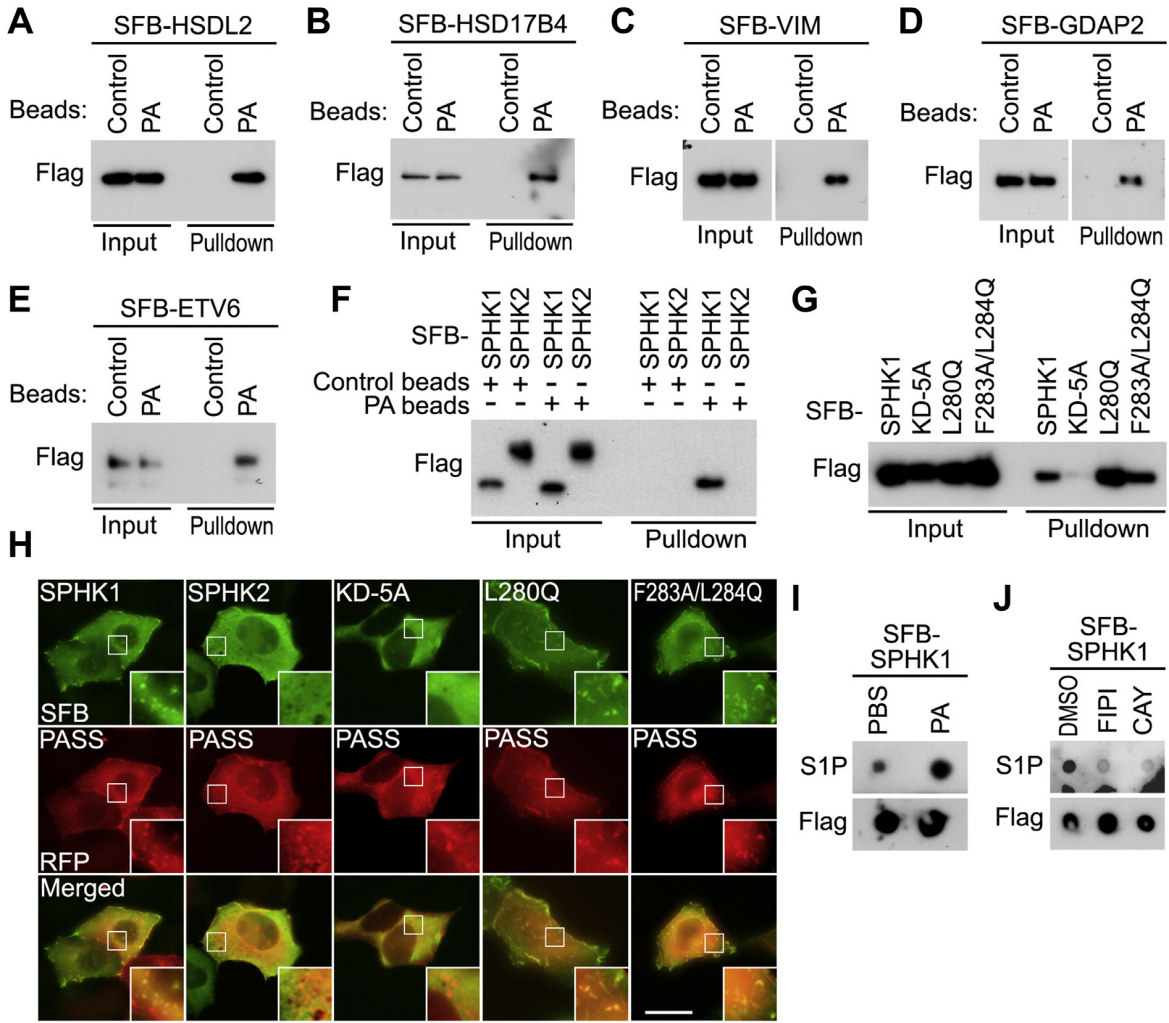
**Analysis of the PA-mediated lipid–protein interaction network uncovers PA as a positive regulator of SPHK1.***A*–*E*, validation of the interaction between PA and its selected HCIPs. Constructs encoding SFB-tagged HSDL2 (*A*), HSD17B4 (*B*), VIM (*C*), GDAP2 (*D*), and ETV6 (*E*) were transfected into HEK293T cells. Pulldown experiments were performed using PA beads. *F*, PA binds SPHK1 but not SPHK2. HEK293T cells were transfected with the constructs encoding the indicated proteins and subjected to pulldown assay using PA beads. *G*, PA does not bind SPHK1 kinase dead mutant. HEK293T cells were transfected with the constructs encoding the indicated proteins and subjected to pulldown assay using PA beads. KD-5A, SPHK1 kinase dead mutant (S165A/G166A/D167A/G168A/L169A). *H*, PA is co-localized with SPHK1 and its two hydrophobic patch mutants but not with its kinase dead mutant or SPHK2. HEK293A cells were transfected with the constructs encoding the indicated proteins and subjected to immunofluorescent staining. Scale bar, 30 μm. *I*, supplementing PA induces SPHK1 kinase activity *in vitro*. SFB-tagged SPHK1 was expressed in HEK293A cells, purified using S protein beads, and subjected to *in vitro* kinase assay in the presence of PA (300 μM), where sphingosine was used as a substrate. The extracted S1P was dotted on nitrocellulose paper and immunoblotted with the indicated antibodies. *J*, inhibition of PA production suppresses SPHK1 kinase activity. SFB-tagged SPHK1 was expressed in HEK293A cells, treated with DMSO, FIPI (30 μM), or CAY10594 (20 μM) overnight, purified using S protein beads, and subjected to *in vitro* kinase assay, where sphingosine was used as a substrate. The extracted S1P was dotted on nitrocellulose paper and immunoblotted with the indicated antibodies. DMSO, dimethylsulfoxide; HCIP, high-confidence interacting protein; PA, phosphatidic acid; PASS, PA sensor protein; PLD, phospholipase D; SFB, S tag-Flag tag-SBP tag; SPHK1, sphingosine kinase 1.

Interestingly, our proteomic study showed that the PA HCIPs have diverse cellular localizations, including cytoplasm, nucleus, cytoskeleton, plasma membrane, ER, peroxisome, mitochondria, endosome, and lysosome (Fig. 3). Based on this finding, we performed the PA-beads pulldown assay and confirmed the association between PA and its HCIPs such as HSDL2 (peroxisome) (Fig. 6*A*), HSD17B4 (peroxisome) (Fig. 6*B*), VIM (cytoskeleton) (Fig. 6*C*), GDAP2 (lysosome) (Fig. 6*D*), and ETV6 (nucleus) (Fig. 6*E*). These results suggest potential roles of PA in the cellular events involving these interacting proteins.

Among the PA HCIPs (Fig. 3 and supplemental Table S3), SPHK1 is a sphingosine kinase, whose product S1P has been discovered as a key signaling molecule involved in various growth-related cellular events such as cell proliferation, survival, migration, and transformation (56). Although the physical interaction between PA and SPHK1 has been previously reported (57), the functional significance underlying this lipid–protein complex formation has not been fully elucidated.

To address it, we further characterized the interaction between PA and SPHK1. As shown in Figure 6*F*, PA specifically interacted with SPHK1 but not SPHK2. Moreover, SPHK1 kinase dead mutant (42, 43, 44) failed to bind PA (Fig. 6*G*), while its two hydrophobic patch mutants (*i.e.*, L280Q and F283A/L284Q), which were previously reported to disrupt the association between SPHK1 and liposome (45), can still bind PA (Fig. 6*G*). We also examined the cellular localization of SPHK1 and PA. As shown in Figure 6*H*, SPHK1 as well as its two hydrophobic patch mutants were co-localized with PA on the punctate structures in the cytoplasm, which are known as Rab5-positive early endosomes and endocytic intermediates (45). In contrast, SPHK2 and SPHK1 kinase dead mutant (KD-5A), which cannot bind PA (Fig. 6, *F* and *G*), failed to localize on the PA-positive punctate structures (Fig. 6*H*), suggesting that PA binding is required in this process.

Because SPHK1 kinase dead mutant (KD-5A) failed to bind PA (Fig. 6*G*), it raised the possibility that PA could regulate SPHK1 kinase activity. To test it, we isolated SPHK1 from the serum starved HEK293A cells, incubated it with PA, and subjected it to *in vitro* kinase assay using sphingosine as a substrate. As shown in Figure 6*I*, PA incubation largely enhanced the ability of SPHK1 to phosphorylate sphingosine to produce S1P. Moreover, treatment with PLD1/2 inhibitors FIPI and CAY10594 significantly reduced the SPHK1-induced S1P production (Fig. 6*J*), suggesting that targeting PA production inhibited SPHK1 kinase activity *in vivo*. Taken together, these data demonstrate PA as a positive regulator of SPHK1, where PA binds SPHK1 and regulates its cellular localization and kinase activity.

## Discussion

In this study, we defined the protein interaction landscape for the human PLD family enzymes and their lipid product PA and identified over 300 HCIPs for them, which greatly expanded our knowledge of this lipid metabolic pathway in diverse signaling events and cellular functions.

As some PLD members comprised similar protein domains (Fig. 1*B*) and showed similar subcellular localizations (Fig. 1*A*), PLD enzymes were found to share some binding partners despite having the unique interacting proteins of their own (Figs. 2 and 3; supplemental Fig. S2), providing us an opportunity to further explore both generic and specific functions/regulations for the PLD enzymes. Indeed, our validation studies not only revealed several specific protein complexes for PLD members such as PLD3–PLD4 (Fig. 4*B*), PLD3–PNPLA6 (Fig. 4*C*), PLD4–RPN2 (Fig. 4*D*), PLD4–TMX3 (Fig. 4*D*), and PLD1–PJA2 (Fig. 5*G*) but also discovered TM9SFs and ANKMY2 as shared binding partners for PLD3/PLD4 (Fig. 4, *A* and *E*–*H*) and PLD1/PLD2/PLD5 (Fig. 5, *A* and *B*), respectively.

Interestingly, our functional studies also connected the PLD family members to several unexpected cellular events. For example, although PLD3 and PLD4 are ER-bound proteins (Fig. 1*A*), their ER-related functions remain largely unknown. Notably, our study uncovered many key players in ER protein quality control as binding partners for PLD3 and PLD4 (Figs. 2*D* and 3), suggesting potential roles of PLD3 and PLD4 in regulating ER stress response. In addition, PLD3 and PLD4 formed a complex with lysosome proteins TM9SFs (Fig. 4, *A* and *E*–*H*), and overexpression of PLD3 induced the contact formation between ER and lysosomes (Fig. 4, *I* and *J*). As PLD3/4 and TM9SFs are transmembrane proteins on ER and lysosomes, respectively, following work will be focused on determining whether their interaction is directly mediated by their cytosolic regions or through additional “ligand factors”. In addition, the signaling contexts as well as the functional significance underlying the PLD3/4–TM9SFs complex formation deserve further investigation. Strikingly, our data also revealed the heterodimerization and homodimerization occurring between PLD3 and PLD4 (Fig. 4*B*). Therefore, it will be highly interesting to elucidate the role of such dimer formation in regulating the ER-related functions of PLD3/4 as mentioned above.

Our proteomic analysis uncovered PJA2 as an E3 ubiquitin ligase for PLD1 (Fig. 5, *G*–*L*), whose loss increased mTOR signaling (Fig. 5*K*). Although PLD1 also functions as a negative regulator of the Hippo pathway (19), we did not observe a dramatic change of YAP phosphorylation or subcellular localization in the PJA2 KO HEK293A cells. This could be explained by the fact that PJA2 deficiency also stabilized MOB1, a key Hippo pathway adaptor, to activate Hippo signaling (41) and compromise the effect as caused by the increased expression of PLD1. Actually, in addition to PLD1 and MOB1, PJA2 has been identified as an E3 ubiquitin ligase for other key signaling proteins, including PKA (58), CDK5R1 (59), KSR1 (60, 61), HIV-1 protein Tat (62), MFHAS1 (63), and TCF/LEF1 (64), highlighting a complex downstream signaling network as controlled by PJA2.

Many studies including ours have revealed PA as a key signaling molecule through its physical interaction with proteins, which prompted us to use a proteomic approach to investigate the PA-associated proteins. Through it, we identified a group of proteins as PA binding proteins (Figs. 3 and 6, *A*–*F*), linking the PLD-PA lipid pathway to additional cellular events and biological processes. Among them, we experimentally confirmed the lipid–protein interaction between PA and SPHK1, which has been previously discovered using different approaches (45, 57). Notably, our functional study further demonstrated PA as a key regulator of SPHK1 to modulate its cellular localization and kinase activity (Fig. 6, *G*–*J*), providing novel mechanistic insights into the regulation of this key lipid kinase. Given the crucial roles of SPHK1 in cell proliferation, survival, migration, differentiation, and cancer development (65, 66), it will be highly important to determine whether these SPHK1-related cellular events also involve the PLD-PA lipid pathway.

Notably, although our proteomic study successfully identified a group of PA-binding proteins, we failed to discover some previously reported ones such as PI4P5K, Raf-1, SHP-1, and mTOR (67). This could be explained by the limitation of the PA-conjugated agarose beads. Based on the information provided by the manufacturer, the PA beads used in our study contain the PA with one C-16 carbon chain and one C-6 carbon chain, where the C-6 chain is modified for agarose bead conjugation. Therefore, such conjugation with agarose beads may affect the binding of PA with some proteins and affect the PA membrane accessibility. In addition, our stringent washing process could also interfere with the capture of some transient and/or weak interacting proteins for PA.

In summary, our proteomic study of the PLDs and PA-centered protein interaction network not only reveals a number of cellular functions/regulations for the PLDs/PA-related signaling events but also generates a comprehensive interactome resource for further characterization of this important lipid metabolic pathway in various biological processes.

## Data Availability

The proteomic data have been deposited in the ProteomeXchange Consortium database (http://proteomecentral.proteomexchange.org/) *via* the PRIDE partner repository (68) with the project identifier PXD029183.

## Supplemental data

This article contains supplemental data.

## Conflict of interest

The authors declare no competing financial interests.

## Supplemental Data

Supplemental Table S1 Complete peptide identification list for human PLDs and PA-based MS experiments.

Supplemental Table S2 Complete prey list for human PLDs and PA-based MS experiments.

Supplemental Table S3 Complete HCIP list for human PLDs and PA-based MS experiments.

Supplemental Table S4 Comparison of the PLDs HCIPs in BioPlex.

Supplemental Table S5 GO analyses of human PLDs and PA-associated HCIPs.

Supplemental Table S6 Sequence information of PJA2 sgRNAs.
